# Field Tests of a Portable MEMS Gravimeter

**DOI:** 10.3390/s17112571

**Published:** 2017-11-08

**Authors:** Richard P. Middlemiss, Steven G. Bramsiepe, Rebecca Douglas, James Hough, Douglas J. Paul, Sheila Rowan, Giles D. Hammond

**Affiliations:** 1 School of Physics and Astronomy, University of Glasgow, Kelvin Building, University Avenue, Glasgow G12 8SU, UK; S.Bramsiepe.1@research.gla.ac.uk (S.G.B.); Rebecca.Douglas@glasgow.ac.uk (R.D.); James.Hough@glasgow.ac.uk (J.H.); Sheila.Rowan@glasgow.ac.uk (S.R.); Giles.Hammond@glasgow.ac.uk (G.D.H.); 2 School of Engineering, University of Glasgow, Rankine Building, Oakfield Avenue, Glasgow G12 8LT, UK; Douglas.Paul@glasgow.ac.uk

**Keywords:** gravity, gravimeter, gravimetry, MEMS

## Abstract

Gravimeters are used to measure density anomalies under the ground. They are applied in many different fields from volcanology to oil and gas exploration, but present commercial systems are costly and massive. A new type of gravity sensor has been developed that utilises the same fabrication methods as those used to make mobile phone accelerometers. In this study, we describe the first results of a field-portable microelectromechanical system (MEMS) gravimeter. The stability of the gravimeter is demonstrated through undertaking a multi-day measurement with a standard deviation of $5.58\times 10^{-6}$ ms${}^{-2}$. It is then demonstrated that a change in gravitational acceleration of $4.5\times 10^{-5}$ ms${}^{-2}$ can be measured as the device is moved between the top and the bottom of a 20.7 m lift shaft with a signal-to-noise ratio (SNR) of 14.25. Finally, the device is demonstrated to be stable in a more harsh environment: a $4.5\times 10^{-4}$ ms${}^{-2}$ gravity variation is measured between the top and bottom of a 275-m hill with an SNR of 15.88. These initial field-tests are an important step towards a chip-sized gravity sensor.

## 1. Introduction

Gravimeters are used to measure variations in the local gravitational acceleration, *g*. Globally, this has an average value of 9.8 ms${}^{-2}$, but it varies by about 0.4% between the equator and the poles [1]. Variations also occur at a smaller scale; accurately measuring these variations both temporally and spatially can provide information about density variations underground. This capability is of use in many different areas: for the hydrocarbon exploration industry [2,3]; the defence industry when searching for hidden tunnels or voids [4,5]; in civil engineering for the investigation of sink holes [6]; for the investigation of magma intrusion in volcanos [7,8,9,10,11]; and they have even been used for unobtrusively finding forgotten crypts at archaeological sites [12].

Excellent commercial gravimeters are available that can measure variations down to the level of 10${}^{-8}$ ms${}^{-2}/\sqrt{\mathrm{Hz}}$ [13] (i.e., variations in gravity one billionth the value of *g* in an integration time of one second). The main drawback of these tools, however, is their high cost and weight. The cutting edge field portable gravimeters weigh over 5 kg and cost over £80 k. If it were possible to drastically reduce the size and cost of gravimeters, then entirely new imaging modalities would become possible. Instead of monitoring single points during a gravity survey, arrays of gravimeters could be networked around the site and set to continuously record. Like a multi-pixel camera, arrays of gravimeters could allow true gravity imaging.

The most promising method by which small and cheap gravimeters could be created is to utilise the fabrication processes used to create silicon mobile phone accelerometers. These silicon chips are known as microelectromechanical systems—or MEMS. MEMS accelerometers in phones cannot measure accelerations below $6\times 10^{-4}$ ms${}^{-2}/\sqrt{\mathrm{Hz}}$, but they are cheap to mass produce and are very small. Such devices consist of a mass on a spring—forming an oscillating system. Below the resonant frequency of the oscillator, there is a constant relationship between the displacement of the mass and the acceleration experienced by the external housing of the frame from which it is suspended. Therefore, by monitoring the relative displacement of the mass, a measurement of the acceleration can be made. Such devices can only measure relative changes in acceleration, not absolute values of *g*.

A few MEMS accelerometers have been created that can achieve measurement sensitivities in the range of 1–20 × 10${}^{-8}$ ms${}^{-2}/\sqrt{\mathrm{Hz}}$ [14,15,16,17,18,19]. Although demonstrating excellent acceleration sensitivity, these devices have not yet demonstrated a long-term stability below mHz levels (despite a desire for this capability [20,21,22]). A useful gravimeter requires stable operation down to 10${}^{-5}$ Hz or lower in order to measure slowly varying geological processes. A MEMS sensor capable of acceleration measurements 40 × 10${}^{-8}$ ms${}^{-2}/\sqrt{\mathrm{Hz}}$ down to 10${}^{-6}$ Hz was demonstrated by the authors [23,24,25,26]. Over a period of six days, this sensor was used to measure the Earth tides—the elastic deformation of the Earth’s crust due to tidal forces.

**Figure 1:**
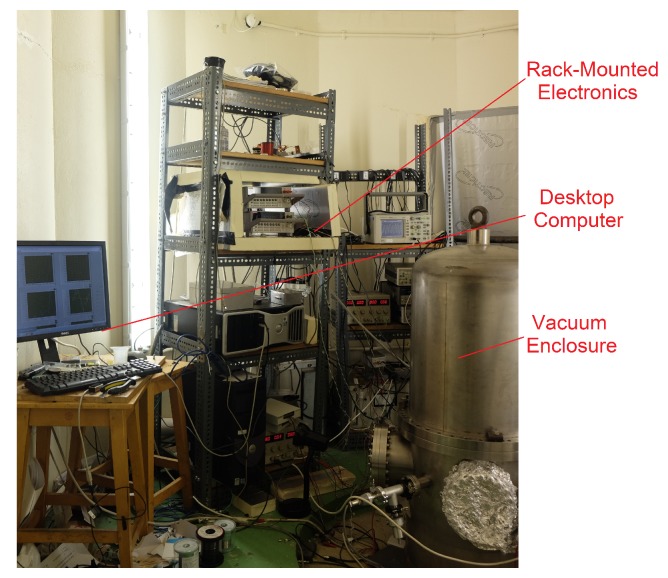
The initial microelectromechanical system (MEMS) sensor [23] was housed within a large vacuum tank and used large, rack-mounted electronics to measure the signal.

**Figure 2:**
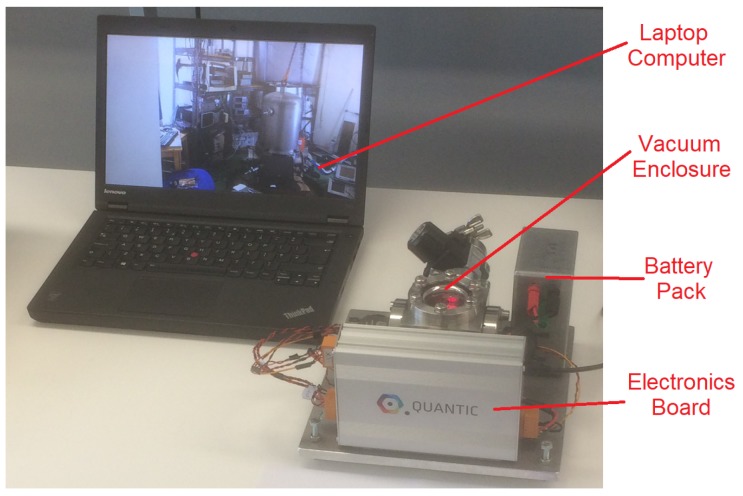
An image of the miniaturised version of the apparatus seen in Figure 1. This device consists of a small vacuum enclosure and an electronics board to control the temperature of the MEMS and process the output signal. Both are mounted on a plate that can be used to adjust the tilt of system.

The sensor developed by the authors (known as *Wee-g*) was initially enclosed within a large vacuum tank, and required multiple rack-mounted pieces of electronic equipment to monitor the data from the MEMS chip (see Figure 1). This apparatus is clearly too large to operate outside of the lab. A field-portable version of this system has been constructed (see Figure 2). The MEMS chip itself, and the optical shadow sensor used to measure the displacement of the mass are enclosed within a small vacuum chamber. An electronics board has also been designed that controls the temperature of the MEMS to variations of a few mK, as well as reading in the displacement signal via a software-based lock-in amplifier. This functionality is dependent on a *dsPIC* microcontroller. The output data can be recorded on an on-board SD card or with an external laptop. The details of the development of this optical sensor and readout circuitry can be seen in the paper by Bramsiepe et al. [27]. The new system now has a footprint of around 30 cm by 30 cm, weighs around 10 kg, and has a power consumption of around 3.2 W. The size, weight and power consumption of the device can all be reduced significantly, but the system as it stands allows hands-on modifications to be made while the field tests are being carried out. Work is underway to enclose the MEMS sensor within a standard MEMS package, shrinking the size of the system by a factor of approximately 10. This article, however, will focus upon the initial trials conducted with the intermediate device pictured in Figure 2. These tests validate the efficacy of the sensor, paving the way for a smaller version in the near future.

## 2. Gravimeter Assembly

**Figure 3:**
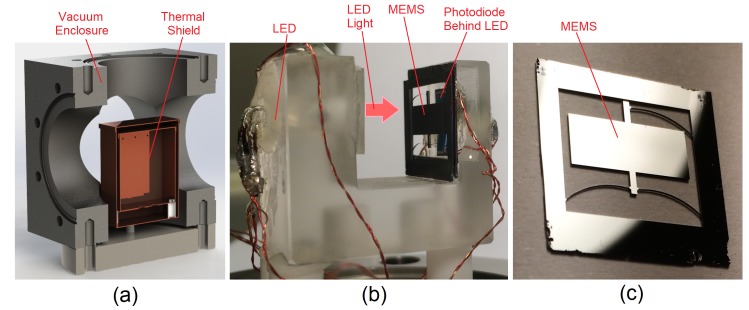
An image of the inside of the vacuum enclosure seen in Figure 2. Image (**a**) is a computer-generated cut-through of the stainless steel vacuum enclosure and the copper thermal shield. Image (**b**) is a photograph of the optical shadow sensor with the MEMS device mounted in its correct position. The light emitting diode (LED) shines light on the photodiode; as the MEMS moves through this light, the change in shadow creates a change in the photodiode output. The wires in this image are used to power the LED, carry the signal from the photodiode, monitor the temperatures of several components, and input power to resistive heaters used to control the temperature of the system to variation of a few mK. Image (**c**) is a close-up photograph of the silicon MEMS device itself: a central mass is suspended from anti-spring flexures.

The newly packaged gravimeter can be seen in Figure 2. To reach this stage, however, some assembly was required. The integral component of the gravimeter is the mass-on-spring. This is the MEMS chip, monolithically etched from silicon using a deep reactive ion etch (DRIE) process [28]. The MEMS oscillator utilises a geometrical anti-spring configuration to ensure a low frequency primary resonance. The lower the frequency, the more the mass will move for a given acceleration, therefore lowering the acceleration noise floor. The optimum acceleration sensitivity is proportional to the square of the resonant frequency (see Equations (1) and (2)). The resonant frequency of the MEMS can be controlled in the fabrication process by altering the thickness of the anti-spring flexures from which the mass is suspended. For the series of experiments discussed in this paper, a MEMS was used that had been fabricated with a resonant frequency of 8.4 Hz (further details at the end of this section). The MEMS was placed in an optical shadow sensor [27] and affixed with wax. Shadow sensors have been used in the Advanced LIGO gravitational wave detector to achieve sub-nm precision measurements [29]. Owing to this extremely good displacement sensitivity, their relative simplicity of design, and their low use of power, this method was a natural means of monitoring the position of the MEMS device in the gravimeter. The shadow sensor is simply comprised of a light emitting diode (LED) pointing at a split photodiode. With the MEMS placed in between these two components, any movement of the shadow cast by the MEMS results in a change in the photodiode output. Two sets of thermometers and heaters were attached to the shadow sensor to enable precise temperature control. This assembly was then enclosed within a copper thermal shield, which could also be temperature controlled using attached heaters and thermometers). The shield and its contents were then placed inside the vacuum enclosure presented in Figure 3, which was then evacuated with a roughing pump to mbarr pressures to thermally isolate the MEMS device. The valve was then closed and the pump was detached from the device. All of these components are mounted upon a metal plate, which can be levelled using three micrometer legs and a tilt-meter. The tilt was monitored using an electrolytic bubble meter, which could measure tilt with a sensitivity of greater than ±2 μRadians. The final device weighed around 10 kg, and was contained within a volume of 30 cm × 30 cm × 30 cm.

The noise floor of the shadow sensor [27] is limited at high frequencies by certain components of the electronics board (specifically an analogue-to-digital converter [ADC]). This noise is white in nature down to sampling frequencies of around $10^{-4}$ Hz. Where the noise is white, averaging can be used to improve the accuracy of the measurement. By averaging for 1000 s, the displacement noise level of the optical sensor has an r.m.s value of 0.6 nm. This noise floor is within an order of magnitude of the shot noise limit, the fundamental limit of this sensing methodology. At lower frequencies (below $10^{-4}$ Hz), the noise of the system is no longer white. The noise in this regime is dominated by fluctuations in intensity and wavelength of the LED. The sensor is sensitive to LED fluctuations because the design does not facilitate ratiometric measurements; if the power fluctuations could be monitored externally, then corresponding fluctuations in the data could be removed. This problem is reduced by ensuring that the proof mass is centered as well as possible between the two segments of the split photodiode, where common-mode power fluctuations are mitigated by the differential measurement.

Given the displacement noise floor of 0.6 nm, the acceleration sensitivity of the device can be calculated using Equation (1):

(1) $$g=\frac{kx}{m}$$

where *k* is the total spring constant of the anti-spring flexures, *x* is the displacement sensitivity, and *m* is the mass of the oscillating mass. The spring constant is given by Equation (2):

(2) $$k=m{\left(2\pi f_{0}\right)}^{2}$$

where $f_{0}$ is the linear resonant frequency of the MEMS. For a displacement noise floor of 0.6 nm, and a resonant frequency of 8.4 Hz, the acceleration noise floor is calculated to be $1.7\times 10^{-6}$ ms${}^{-2}$ (over an integration time of 1000 s). As mentioned in the introduction, commercial gravimeters can achieve sensitivities as low as a $1\times 10^{-8}$ ms${}^{-2}$. This device, therefore, would be improved with the use of a lower frequency MEMS (or a more sensitive displacement sensor). For previous measurements [23] a MEMS with a resonant frequency of 2.2 Hz was used. If such a MEMS device were placed in the current sensor, an acceleration noise floor of $1.1\times 10^{-7}$ ms${}^{-2}$ would be possible. This would allow the observation of the Earth tides, which have a peak amplitude of $3\times 10^{-6}$ ms${}^{-2}$. This sensitivity level would also put the device within an order of magnitude of the top commercial gravimeters, and easily within the range that would make the device useful for the applications described above. An 8.4 Hz device was selected for these first field tests because the stress in the flexures is lower than that of a 2.2 Hz MEMS, allowing greater robustness for the initial tests. The acceleration sensitivity of $1.7\times 10^{-6}$ ms${}^{-2}$ is perfectly sufficient to demonstrate the operation of the gravimeter in the field, the goal of this series of experiments. After performing these measurements, it is intended to introduce lower frequency devices to incrementally improve the sensitivity of the gravimeter.

## 3. Noise and Stability Tests

Before conducting tests of the instrument’s ability to monitor variations in gravitational acceleration, it was first necessary to test the performance of the device when no changes in acceleration were expected. No measurements could be trusted if it were not known that the system would remain stable whilst in one location.

**Figure 4:**
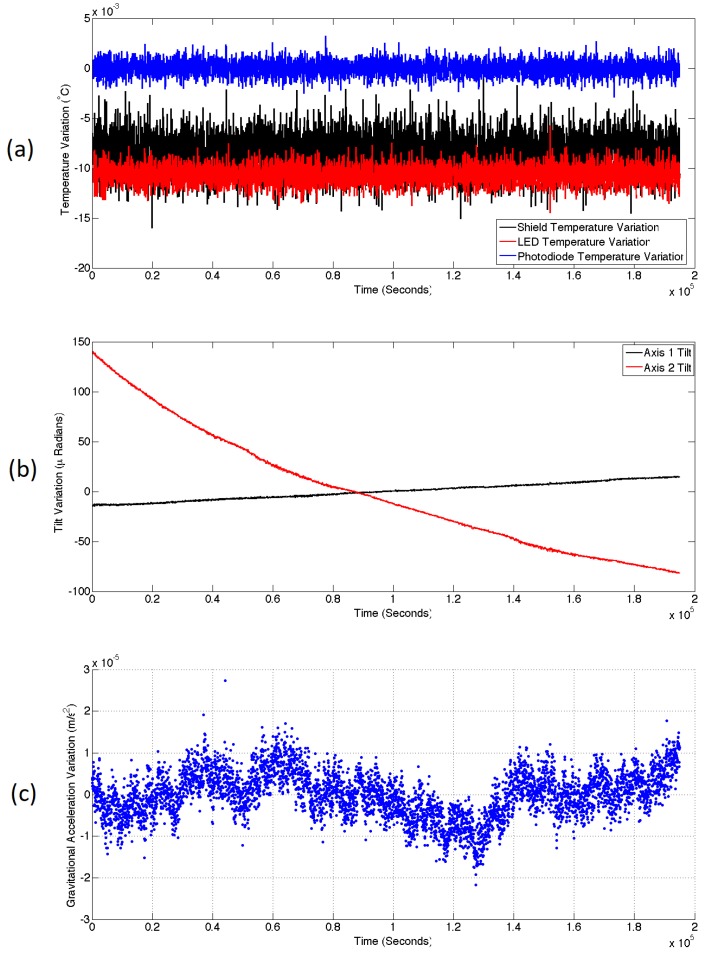
The noise performance of the MEMS gravimeter over a period of 54 h. (**a**) Shows the normalised temperature variation of the shield, LED and Photodiode over this period. These temperatures were stabilised using a proportional integral derivative (PID) control mechanism. (**b**) Shows the tilt of two orthogonal axes of the device. The large variation of axis 2 was caused by the sinking of the device into the linoleum of the laboratory floor. (**c**) Shows the acceleration output of the device over this period. A multiple linear regression has been performed on this data to remove the effect of the tilt and a residual linear drift. It is not expected that the Earth tides could be observed with this noise level, but if a lower frequency device were inserted into the sensor then such an observation could be made.

The gravimeter was left in the basement of the building for a period of 54 h. The temperatures of the copper shield, the LED and the photodiode were all controlled using three independent proportional integral derivative (PID) control systems [27]. All three temperatures remained stable during this period at a level above ambient room temperature. The normalised temperature variations for all three measurements are displayed in Figure 4a. The standard deviation of the shield, LED, and photodiode temperatures were $1.96\times 10^{-3}$ ${}^{\circ }$C, $9.07\times 10^{-4}$ ${}^{\circ }$C, and $7.68\times 10^{-4}$ ${}^{\circ }$C respectively. The sampling rate for these measurements was 2.13 s (this is also the case for all of the data references in this article used to calculate standard deviation measurements). It was known that the most temperature sensitive component was the LED, which would produce a change in the gravimeter output of $1.59\times 10^{-6}$ ms${}^{-2}$/mK. Given the variation of the temperatures, it was not expected that temperature variations would cause noticeable changes to the gravimeter output.

Although the temperatures did not cause any variation in the gravimeter output, it was noticed in that there was a correlation between the output and the tilt of the device. The tilt data of two orthogonal axes is displayed in Figure 4b. Axis 1 (black) is the axis parallel to the plane of the MEMS chip, and axis 2 (red) is the axis perpendicular to the MEMS chip. It was known that variations in the parallel plane caused output changes of $3.64\times 10^{-6}$ ms${}^{-2}$/μRad. For the perpendicular axis, this calibration was $6.26\times 10^{-7}$ ms${}^{-2}$/μRad. The system had tilted markedly in axis 2 overnight as it sunk into the linoleum of the laboratory. A multiple linear regression of the data [23] was therefore carried out to remove the effect of these tilt correlations, as well as a residual linear drift with a regression coefficient of $-2.64\times 10^{-9}$ ms${}^{-2}$/s (or $-2.28\times 10^{-4}$ ms${}^{-2}$/day). The resulting data is displayed in Figure 4c. This time series demonstrates the long-term noise performance of the sensor left in a single location. This series has a standard deviation of $5.58\times 10^{-6}$ ms${}^{-2}$. The long period variations in this data are not correlated with temperature and are thought to be due to power or wavelength fluctuations of the LED.

**Figure 5:**
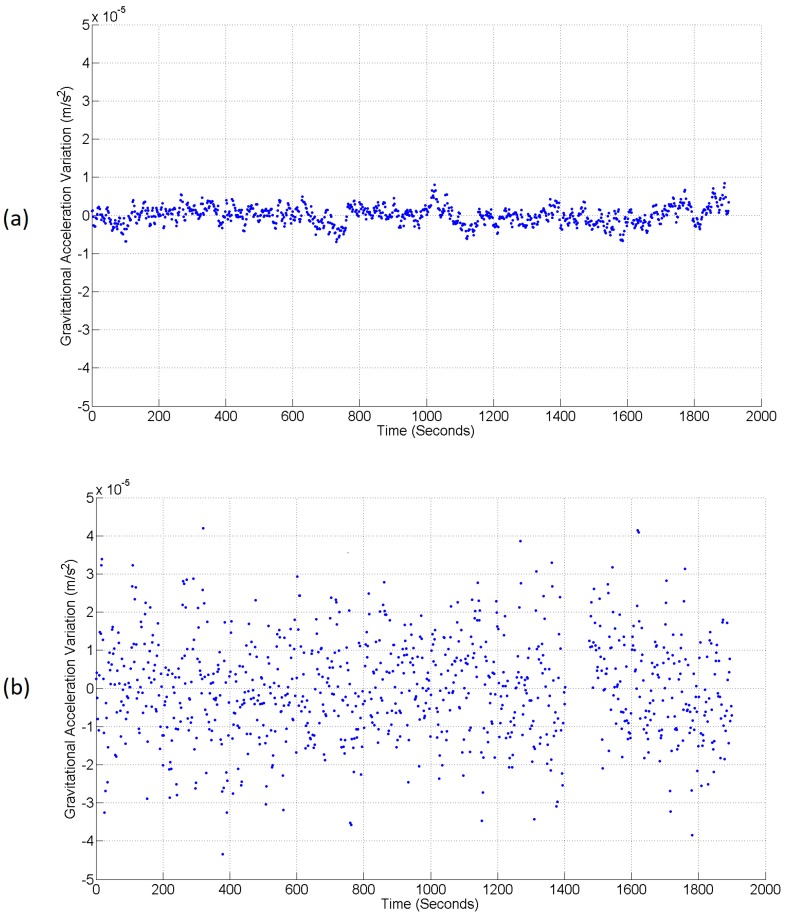
Two time series demonstrating the noise level of the gravimeter inside and outside ((**a**) and (**b**) respectively). It can be seen that the noise level increases significantly outside the confines of the laboratory (with a standard deviation of $1.31\times 10^{-5}$ ms${}^{-2}$ compared to $2.40\times 10^{-6}$ ms${}^{-2}$ inside; each of these was calculated with an integration time of 2.13 s). The increased noise is due to the harsher environmental conditions: wind buffeting and larger temperature gradients. A short section of data has been removed from this series at 1400 s, where a loose connection was fixed; spiking the data.

The next test was to compare the noise level of the device between an inside and an outside test location. This test was carried out over a shorter timescale due to the logistical barriers to leaving the system outside overnight. Figure 5a demonstrates the first 1900 s of the laboratory data already displayed in Figure 4c. Over this short period, the series has a standard deviation of $2.40\times 10^{-6}$ ms${}^{-2}$. Figure 5b is the data from a corresponding measurement conducted outside the building. Outside, the system had to contend with the effect of wind and a larger variation in temperature. Although the PID controllers were able to counteract these larger variations, the noise on the shield, LED and photodiode temperature measurements were still approximately an order of magnitude larger: $3.81\times 10^{-2}$ ${}^{\circ }$C, $2.75\times 10^{-3}$ ${}^{\circ }$C, and $2.51\times 10^{-2}$ ${}^{\circ }$C respectively. Another linear regression was carried out to remove variations in the output caused by coupling of the tilt and the linear drift. It is this resulting data that can be observed in Figure 5b. Due to the increased temperature noise, the standard deviation of the gravimeter data was also noisier than in the inside test; it was measured to be $1.31\times 10^{-5}$ ms${}^{-2}$. It is expected that the temperatures can be better controlled (and therefore the gravimeter noise reduced) by changing the operational amplifiers responsible for driving the PID heaters. The operational amplifiers used to conduct this experiment were limited in how much current they could draw. Replacements capable of drawing a greater current would allow a greater power to be provided to the heaters, thus providing an ability to maintain the internal temperature during more extreme swings in external temperature. Crucially, the outside noise level is still white, meaning that averaging will still be possible; it will just take longer to reach the same sensitivity observed inside.

## 4. Altitude Tests

To demonstrate the efficacy of the MEMS gravimeter, the change in gravity with altitude was measured. Most simply, the value of *g* gets smaller with increasing distance from the centre of the planet. Using Newton’s universal law of gravitation, the change in *g* as a function of altitude *z* can simply be expressed as [1]:

(3) $$\Delta g=-\frac{2GM_{E}\Delta z}{R_{E}^{3}}=-\frac{2g\Delta z}{R_{E}}\;{\mathrm{ms}}^{-2}$$

where *G* is the gravitational constant, $M_{E}$ is the mass of the Earth, and $R_{E}$ is the radius of the Earth. This is known as the Free-Air Effect, and it corresponds to a variation of $3.1\times 10^{-6}$ ms${}^{-2}$ per vertical metre away from the Earth’s surface.

The Free-Air Effect can be used to calculate the gravitational variation only if there is nothing massive between the two measurement points. Many gravity measurements are carried out on high terrain; in this case, there is a large volume of massive material between the gravimeter and the lower measurement point. This massive material will create a gravitational acceleration with a force vector in the opposite direction to the Free-Air Effect. This phenomenon is known as the *Bouguer Anomaly*. It normally reduces the Free-Air variation by around one-third, but its magnitude varies according to topology, and rock density. A simple analytical solution for the Bouguer Anomaly can be found by assuming that the upper survey point is located on an infinitely wide slab of rock, with a thickness, *H*, and a density, ρ. In this circumstance, the Bouguer Anomaly can be expressed as [1]:

(4) $$\Delta g=+2\pi \rho GH\;{\mathrm{ms}}^{-2}$$

Given that the Bouguer Anomaly is so dependent on topology and rock density, it is very difficult to make accurate predictions of its magnitude. It is more common to use a gravimeter to measure the Bouguer Anomaly to infer details about rock density, than it is to carry out the reverse process.

### 4.1. Lift Tests

**Figure 6:**
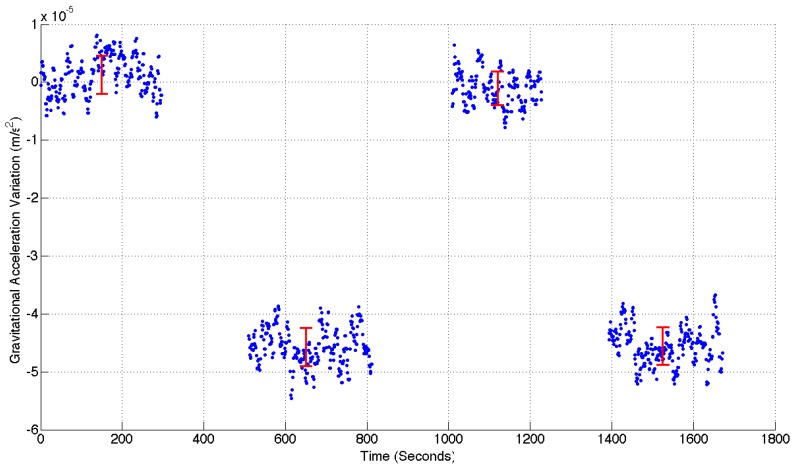
The change in gravitational acceleration observed between the bottom and top of a 20.73 m lift shaft, with two measurements conducted in each position. The zero point of the data has been set as the level measured at the bottom of the lift shaft. An acceleration change of $4.51\times 10^{-5}$ ms${}^{-2}$ is observed between the top and bottom of the building. The data has been cut in between each of the measurements since this just shows the inertial acceleration caused by the motion of the lift. The red data points are the mean acceleration value of each measurement location. The error bars of these points demonstrate the standard deviation of each respective series.

The first altitude test conducted with the MEMS gravimeter was to place the instrument in the lift of the building. The gravimeter was placed on the floor of the lift and levelled using the micrometer legs on its base. The base needed to be levelled because tilt of the MEMS produces a parasitic measurement of gravitational acceleration. This is due to Einstein’s equivalence principle, which states that inertial and gravitational accelerations are indistinguishable. The micrometer legs could be used to level the system to within ±2 μRadians. The levelling process would excite the oscillator so it would be left for around five minutes to settle before a stable measurement could be taken. Measurements were taken for approximately five minutes before taking the lift to the top/bottom of the building and repeating this process. Four such measurements were taken, starting in the basement of the building. The lift has a vertical range of 20.73 m. Assuming that the building has nominally zero density, the gravity variation expected would be the direct result of the Free-Air Effect: $6.44\times 10^{-5}$ ms${}^{-2}$. The experimental data from this experiment can be observed in Figure 6.

The gaps in the data in Figure 6 correspond to the times during which the oscillator was excited. The data has also been regressed in the manner described in the previous section. This measurement demonstrates a signal-to-noise ratio (SNR) of 14.25. This value was calculated by taking the ratio of the mean acceleration variation between the top and bottom of the lift ($4.51\times 10^{-5}$ ms${}^{-2}$), and the mean standard deviation of all four measurement locations ($3.16\times 10^{-6}$ ms${}^{-2}$). The red data points in this graph represent the average of the values at each measurement location, with error bars determined by the standard deviation of each series. A clear variation in gravitational acceleration has been observed. Since the first measurement was taken at the bottom of the building, it is also clear that the sign of this signal is correct. It is apparent, however, that the variation in gravitational acceleration does not exactly match the prediction of $6.44\times 10^{-5}$ ms${}^{-2}$ based on the Free-Air Effect alone. The measured signal variation of $4.51\times 10^{-5}$ ms${}^{-2}$ is clearly smaller than the calculated value. It is expected that this discrepancy can be accounted for by the fact that the lower three levels (approximately nine meters) of the lift shaft lie beneath ground level. The surrounding soil and rock will therefore reduce the size of the expected signal. This effect is two-fold: the amplitude of the measurements taken in the basement will be reduced because the ground above it will create a small upward gravitational force, and the amplitude of measurements taken at the top of the building will be increased because the ground below it will create an additional downward force (a Bouguer Anomaly). The net effect of this topology is to reduce the overall difference in signal observed between the top and bottom of the lift shaft. Based on the rough topology of the ground surrounding the building, an estimation of the signal reduction was made. In the basement location, the upward acceleration could lie between $2\times 10^{-6}$ ms${}^{-2}$ and $3\times 10^{-6}$ ms${}^{-2}$ (assuming a range in ground density of 2000 kg/m${}^{3}$ [soil] to 3000 kg/m${}^{3}$ [Basalt]). In the upper location, the downward acceleration due to the Bouguer Anomaly could lie between $7\times 10^{-6}$ ms${}^{-2}$ and $1.1\times 10^{-5}$ ms${}^{-2}$ (using the same estimated range in ground density). This rough modelling suggests that the measured range in acceleration between the top and bottom of the building could easily be reduced by around $1.4\times 10^{-5}$ ms${}^{-2}$. This reduction would bring the expected signal close to the experimental value. The topology of the ground around and outside the building is difficult to ascertain, however, and the density of the made-ground is also hard to estimate; so it is hard to make anything other than an estimate of the expected signal. It was not possible to use a commercial gravimeter to check the Bouguer Anomaly, so instead another test was carried out over a larger vertical baseline. This is the topic of the following sub-section.

### 4.2. Hill Tests

**Figure 7:**
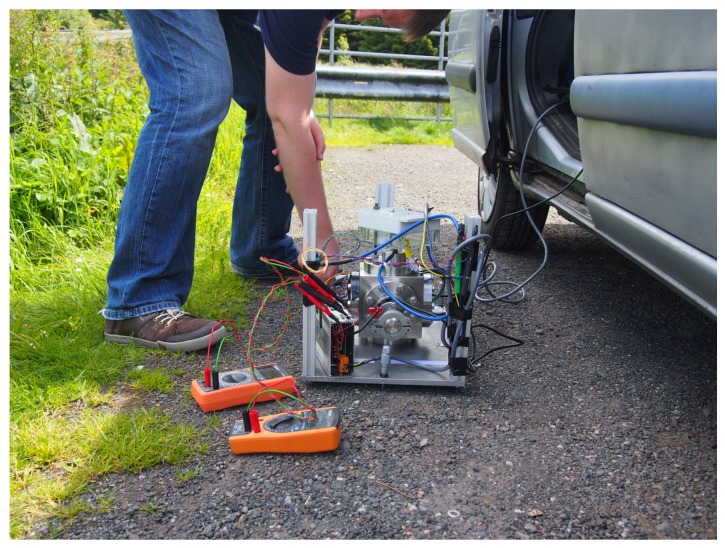
An image of the gravity measurement being conducted at the top of the hill. The user can be seen levelling the platform before the data is recorded.

**Figure 8:**
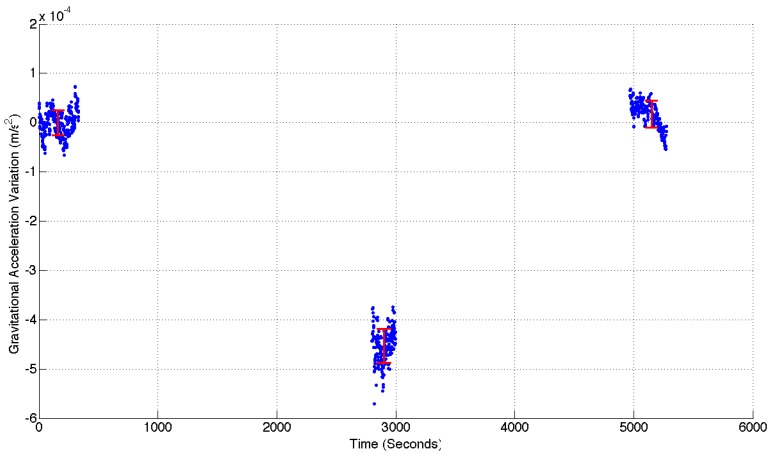
The data recorded on the field test up the Campsie hills. Three measurements can be observed in the graph: one at the base of the hill, one at the top, and a final measurement in the original location. The data has been zeroed at the value measured at the bottom of the hill. The red data points and error bars at each measurement location correspond to the mean value, and the standard deviation respectively. An acceleration variation of $4.62\times 10^{-4}$ ms${}^{-2}$ is demonstrated. This value aligns with the estimations made for the Free-Air Effect and Bouguer Anomaly.

The next test to which the gravimeter was subjected was a genuine field test. The device was driven to the base of a local hill (the *Campsie* range to the north of Glasgow). At the base of the hill, the gravimeter was set up in the same manner as described in the previous sub-section (see Figure 7). The system was levelled and a short time was allowed for the oscillating system to settle. Once a measurement had been made, the gravimeter was driven to the top of the hill (an altitude change of 275 m) and another set of data recorded. A final measurement was made in the same location as the first. The gravimeter ran continuously through these three measurements, and the corresponding data (with the travel and settling times removed) can be observed in Figure 8.

The data presented in Figure 8 demonstrates an SNR of 15.88. This value was calculated by taking the ratio of the mean acceleration variation between the top and bottom of the hill ($4.62\times 10^{-4}$ ms${}^{-2}$), and the mean standard deviation of all three measurement locations ($2.91\times 10^{-5}$ ms${}^{-2}$). If the upper measurement had been made while hovering in free air, the expected signal would be $8.55\times 10^{-4}$ ms${}^{-2}$ (from Equation (3)). This was clearly not the case, however, so some estimate of the Bouguer Anomaly had to be made. Taking the infinite slab model described by Equation (4), and using a rock density of 3000 kg/m${}^{3}$, one can calculate a new total signal strength of $5.8\times 10^{-4}$ ms${}^{-2}$. Although the density of the Campsie range is expected to be around 3000 kg/m${}^{3}$ due to its predominantly basaltic composition [30], this number could vary between 2700 kg/m${}^{3}$ and 3300 kg/m${}^{3}$. The topology of the measurement locations could also alter the size of the Bouguer anomaly. The difference between the Bouguer Anomaly of the infinite slab model compared to a pyramidal structure is about 40%. These unknown variances can be used to calculate a range in which the actual Bouguer Anomaly is likely to lie. Subtracting the maximum and minimum anomalies from the Free-Air total gives an acceleration range between $4.74\times 10^{-4}$ ms${}^{-2}$ and $60.5\times 10^{-4}$ ms${}^{-2}$. The experimental data in Figure 8 lies just beyond the lower limit of this range.

## 5. Conclusions

In this paper, a practical MEMS gravimeter has been demonstrated working outside of the laboratory. The results show that it has an acceleration sensitivity of $1.31\times 10^{-5}$ ms${}^{-2}$ when outside, although this could easily be improved upon by the use of a lower frequency MEMS chip, and by using more powerful operational amplifiers in the temperature control circuitry. The device’s ability to measure altitude variations both inside and outside has clearly been shown by experiments conducted within a lift-shaft, and up and down a local hill. Further characterisation of the system is required by conducting side-by-side tests next to a commercial gravimeter, but the measurements presented in this paper demonstrate that the device is a stepping-stone towards a useful field gravimeter. Once this device is packaged within a standard MEMS enclosure, it will be significantly smaller and lighter than any commercially available gravimeter currently available.

## Abbreviations

The following abbreviations are used in this manuscript:

MEMS	Microelectromechanical System
SNR	Signal-to-Noise Ratio
DRIE	Deep Reactive Ion Etch
LED	Light Emitting Diode
ADC	Analogue-to-Digital Converter
PID	Proportional Integral Derivative
